# Production of Fucoxanthin from *Phaeodactylum tricornutum* Using High Performance Countercurrent Chromatography Retaining Its FOXO3 Nuclear Translocation-Inducing Effect

**DOI:** 10.3390/md19090517

**Published:** 2021-09-11

**Authors:** Daniela Bárcenas-Pérez, Antonín Střížek, Pavel Hrouzek, Jiří Kopecký, Marta Barradas, Arantzazu Sierra-Ramirez, Pablo J. Fernandez-Marcos, José Cheel

**Affiliations:** 1 Laboratory of Algal Biotechnology—Centre ALGATECH, Institute of Microbiology of the Czech Academy of Sciences, Opatovický Mlýn, 379 81 Třeboň, Czech Republic; barcenas@alga.cz (D.B.-P.); strizek@alga.cz (A.S.); hrouzek@alga.cz (P.H.); kopecky@alga.cz (J.K.); 2 Faculty of Science, University of South Bohemia, Branišovská 1760, 370 05 České Budějovice, Czech Republic; 3 Faculty of Science, Department of Ecology, Charles University, Viničná 7, 128 44 Prague 2, Czech Republic; 4 Metabolic Syndrome Group—BIOPROMET, Madrid Institute for Advanced Studies—IMDEA Food, CEI UAM+CSIC, 28049 Madrid, Spain; marta.barradas@imdea.org (M.B.); aranzazu.sierra@imdea.org (A.S.-R.); pablojose.fernandez@imdea.org (P.J.F.-M.)

**Keywords:** fucoxanthin, *Phaeodactylum tricornutum*, high performance countercurrent chromatography (HPCCC), countercurrent chromatography (CCC), centrifugal partition chromatography (CPC)

## Abstract

*Phaeodactylum tricornutum* is a rich source of fucoxanthin, a carotenoid with several health benefits. In the present study, high performance countercurrent chromatography (HPCCC) was used to isolate fucoxanthin from an extract of *P. tricornutum*. A multiple sequential injection HPCCC method was developed combining two elution modes (reverse phase and extrusion). The lower phase of a biphasic solvent system (*n*-heptane, ethyl acetate, ethanol and water, ratio 5/5/6/3, *v*/*v/v/v*) was used as the mobile phase, while the upper phase was the stationary phase. Ten consecutive sample injections (240 mg of extract each) were performed leading to the separation of 38 mg fucoxanthin with purity of 97% and a recovery of 98%. The process throughput was 0.189 g/h, while the efficiency per gram of fucoxanthin was 0.003 g/h. Environmental risk and general process evaluation factors were used for assessment of the developed separation method and compared with existing fucoxanthin liquid-liquid isolation methods. The isolated fucoxanthin retained its well-described ability to induce nuclear translocation of transcription factor FOXO3. Overall, the developed isolation method may represent a useful model to produce biologically active fucoxanthin from diatom biomass.

## 1. Introduction

**Figure 1:**
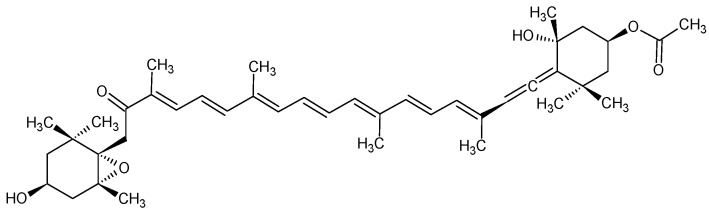
Chemical structure of fucoxanthin.

Fucoxanthin (Figure 1) is an orange carotenoid found in brown seaweeds and some classes of microalgae, especially diatoms [1]. Unlike other carotenoids found in nature, fucoxanthin has a unique molecular structure composed of an unusual allenic bond, a 5,6-monoepoxide and nine conjugated double bounds [2]. This carotenoid has been mainly isolated from seaweeds and widely investigated for its biological properties [2,3]. Its benefits include anti-obesity [4,5,6,7], anti-diabetic [8], anti-inflammatory [9,10,11], anti-cancer [12,13,14,15] and antioxidant [3,16,17,18,19,20] effects. Moreover, fucoxanthin has been previously described to induce nuclear translocation of the transcription factor FOXO3, which inhibits the fibronectin and collagen IV expression as well as oxidative stress, resulting in the reduction of fibrosis in diabetic nephropathy [21,22]. Commercial products containing brown algae-sourced fucoxanthin which are mainly used for weight and fat control include Solaray Fucoxanthin from SolarayVR, fucoTHIN from Garden of Life [23] and ThinOgen Fucoxanthin from BGG [24]. Only one product (FucoVitalTM from Algatech, Ketura) containing fucoxanthin from the diatom *Phaeodactylum tricornutum* is commercially available [23].

Diatom microalgae have gained attention as a valuable source of fucoxanthin since its biomass content can be enriched up to ten times more than for macroalgae [3]. Among diatoms, *P. tricornutum* “the model diatom” is one of the most studied species. Until now, most of the efforts devoted to exploit *P. tricornutum* as a source of fucoxanthin have been mainly focused on culture optimization, reaching fucoxanthin contents up to 16 mg [25] and 26 mg [26] per gram dry weight. Fucoxanthin has an increasing demand in nutraceutical, cosmetic and food industry sectors and its total global market size is expected to reach USD 107.4 million in the period from 2020 to 2025 [27].

So far, the methods reported for the isolation of fucoxanthin from algae have required the use of liquid-solid chromatography [25,28,29,30,31,32,33], which includes multi-step procedures. Fucoxanthin-enriched extracts have been also produced through enzyme-assisted extraction followed by co-solvent extraction [34], aqueous two-phase systems (ATPS) extraction system [35], supercritical CO_2_ extraction [36], pressurized liquid extraction [37,38], pressurized subcritical extraction [26] and subcritical fluid extraction [1]. Despite the aforementioned efforts for obtaining fucoxanthin from algal biomass, it has been noted that low cost, simple and scalable isolation methods still require to be developed [23,39]. In these circumstances, liquid-liquid chromatography such as countercurrent chromatography (CCC) is able to play an essential role, as it takes advantage of its liquid stationary phase. CCC does not use any solid support and the stationary and the mobile phases are liquids [40]. The stationary phase is immobilized in the column by means of a centrifugal force field generated by the column spinning, whereas the mobile phase is pumped through the column. The separation of target compounds from a mixture is based on the difference in their partitioning between the two immiscible phases. Given that this technology lacks solid support, it has many advantages over the solid chromatographic techniques including large sample loading capacity, low risk of irreversible adsorption and sample denaturation, high sample recovery, low consumption of solvents and great operational versatility, since the roles of mobile and stationary phases can be exchanged during the chromatographic operation [40,41,42]. The capacity of countercurrent chromatography for obtaining valuable compounds from microalgae biomass has been widely demonstrated [42,43,44,45,46,47,48] and its application at industrial level is already a reality [49]. High-speed countercurrent chromatography (HSCCC) has been used for the isolation of fucoxanthin from edible brown macroalgae species such as *Laminaria japonica*, *Undaria pinnatifida* and *Sargassum fusiforme* [50] applying a single sample injection method. Centrifugal partition chromatography (CPC), another variant of liquid-liquid chromatography, was also used in two steps to isolate fucoxanthin from the macroalgae *Eisenia bicyclis* [51] and in one step followed by flash chromatography to produce fucoxanthin from the microalgae *Tisochrysis lutea* [52]. The present study reports, for the first time, an efficient and scalable HPCCC isolation method to obtain fucoxanthin from the diatom *P. tricornutum* using a multiple sequential-injection separation strategy. The developed method does not need to align with another chromatographic technique to achieve the desired purity. The biomass extraction and HPCCC isolation process were unified using the same solvent system to improve the fucoxanthin final recovery. The isolated compound was found to maintain its described bioactivity as inductor of nuclear translocation of the transcription factor FOXO3.

## 2. Results and Discussion

### 2.1. Extract Preparation

**Figure 2:**
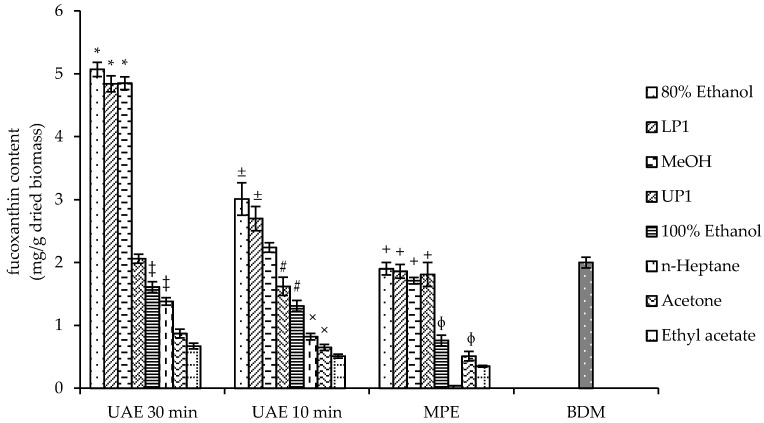
Extraction optimization of fucoxanthin from dried biomass of *Phaeodactylum tricornutum*. Data are means ± SD (n = 3). Bars values with the same symbols (*,‡,±,#,×,+,ɸ) within the same extraction treatment are not significantly different from each other (Tukey’s test, *p* < 0.05). Ultrasound assisted extraction (UAE), mortar and pestle-assisted extraction (MPE), lower phase (LP1) and upper phase (UP1) of the selected biphasic solvent system, Bligh–Dyer method (BDM).

In order to achieve a high recovery of target compounds, the isolation process should be preceded by an efficient extraction method. Generally, the solvent used for obtaining biomass extracts is not the same to that used for the isolation work, since different techniques are applied. Unifying the chemical nature of the solvent used in biomass extraction and isolation could favor the recovery of the target compound, as recently described [45]. In the present study, the upper (UP1) and lower (LP1) phases used for the HPCCC isolation of fucoxanthin (Section 2.2) were tested for their capacity to produce a fucoxanthin-enriched extract from *P. tricornutum* biomass. These liquid phases were compared with other solvents generally used to generate microalgae extracts (Figure 2). Ultrasound assisted extraction (UAE) for 10 and 30 min and mortar and pestle-assisted extraction (MPE) were applied as extraction methods. The results (Figure 2) showed that the fucoxanthin yield was highly dependent on the solvent and extraction method. LP1 and 80% ethanol led to similar fucoxanthin recoveries in all experiments, but the highest values were obtained by using UAE for 30 min. In previous investigations [25,32], 100% ethanol was found to lead to the best extraction yield. To determine the extraction efficiency of LP1 in this study, the Bligh–Dyer method was used as a reference method, as described in Section 3.2. In Figure 2, the contents of fucoxanthin in the extracts produced using UAE for 30 min with LP1 and Bligh–Dyer methods were 4.84 mg/g and 2 mg/g, respectively. Therefore, LP1 showed extraction efficiency over 100%. It is conceivable to assume that the major components in the upper (UP1) and lower (LP1) phases are *n*-heptane and water; respectively, with ethanol and ethyl acetate distributed variably between the two liquid phases. Using LP1 as the solvent for biomass extraction allows a selective extraction that excludes highly lipophilic impurities and, thus, benefits its further isolation. Therefore, the extraction of *P. tricornutum* biomass with LP1 under UAE for 30 min was selected for the large-scale biomass extraction. Using these conditions, an amount of 10 g of dried *P. tricornutum* biomass was extracted with 1.2 L of LP1 affording 3.56 g of dried extract, which was used for the HPCCC isolation of fucoxanthin.

### 2.2. Development and Optimization of HPCCC Separation at Analytical Scale and Scale-Up to Semi-Prep Column 

Different biphasic solvent systems composed of *n*-heptane, ethyl acetate, ethanol and water at different proportions were prepared and investigated for their capacity to be used in the isolation of fucoxanthin from extract of *P. tricornutum* biomass (Table 1). An ideal solvent system has to meet some basic requirements. Firstly, it has to provide a proper *K* value (0.5 ≤ *K* ≤ 3.5) [43,53] that permits the separation of the target compound between the two immiscible phases of the selected biphasic solvent system. Secondly, it has to retain enough stationary phase within the HPCCC column by providing a proper density difference (>0.08 g/mL) between its two immiscible liquid phases [46,54] and a short settling time (t < 30 s) [40]. From the Table 1, the system 1 was selected for the isolation of fucoxanthin and thus transferred to the analytical coil (24 mL) of the HPCCC equipment for the optimization of the isolation process in order to maximize the throughput and efficiency.

**Figure 3:**
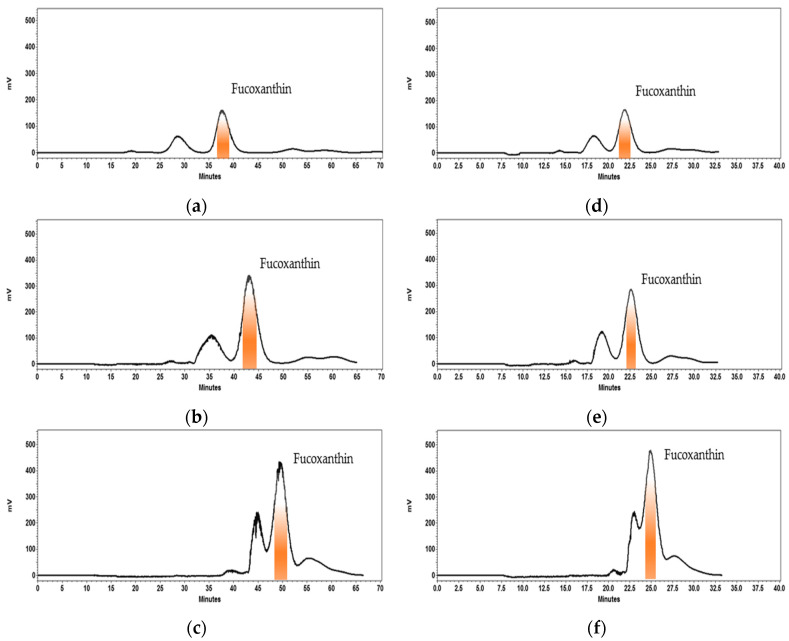
High performance countercurrent chromatography (HPCCC) optimization using different sample loadings and flow rates of mobile phase for obtaining fucoxanthin from *Phaeodactylum tricornutum* extract. (**a**) 20 mg at 0.5 mL/min. (**b**) 40 mg at 0.5 mL/min. (**c**) 60 mg at 0.5 mL/min. (**d**) 20 mg at 1.0 mL/min. (**e**) 40 mg at 1.0 mL/min. (**f**) 60 mg at 1.0 mL/min. The samples were dissolved in 0.5 mL of mobile phase (0.5 mL sample loop). Biphasic solvent system: System 1, mixture of *n*-heptane, ethyl acetate, ethanol and water (ratio 5:5:6:3, *v*/*v/v/v*). Elution mode: Reverse, the mobile phase is the lower phase of the system 1. Column temperature: 30 °C. Detection: 450 nm.

It has been well established that a high retention of the stationary phase leads to a good resolution in countercurrent chromatography [55]. Given that flow rate and sample loading can affect the retention of the stationary phase, these parameters were optimized in the current study. The optimization studies were performed using sample loadings from 20 to 60 mg and mobile phase flow rates of 0.5 and 1.0 mL/min using the analytical coil (24 mL) of the HPCCC equipment. The column rotational speed and loop volume were at fixed 1600 rpm and 0.5 mL, respectively, operating at 30 °C. This process was performed in reverse elution mode, which means that the lower phase of the selected biphasic solvent system was used as the mobile phase, while the upper phase was the stationary phase. It was observed that fucoxanthin was well separated with sample loadings of 20 and 40 mg at mobile phase flow rates of both 0.5 and 1 mL/min (Figure 3a,b,d,e). These operating conditions permitted a good retention of stationary phase within the column (Table 2), which was estimated using equation 1. The highest fucoxanthin purity was achieved at a flow rate of 0.5 mL/min and when 20 or 40 mg of sample was injected (Table 2). Finally, a sample loading of 40 mg was chosen as the optimal value, as it would favor the process throughput. 

**Figure 4:**
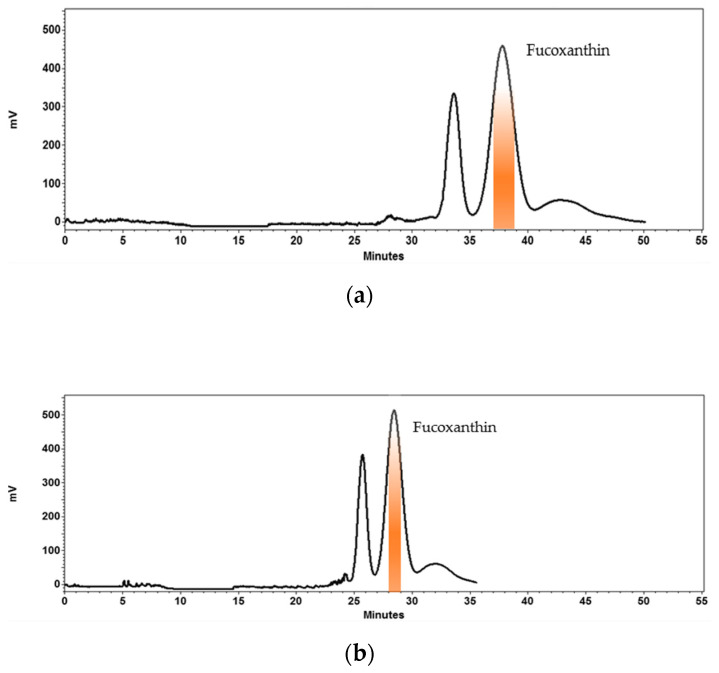
Scale-up of high performance countercurrent chromatography (HPCCC) method to obtain fucoxanthin from *Phaeodactylum tricornutum* extract. Biphasic solvent system: System 1, mixture of *n*-heptane, ethyl acetate, ethanol and water (ratio 5:5:6:3, *v*/*v*/*v*/*v*). Loading per injection: 240 mg of *Phaeodactylum tricornutum* extract dissolved in 3 mL of mobile phase (3 mL sample loop). (**a**) 3 mL/min of flow rate of the mobile phase. (**b**) 4 mL/min of flow rate of the mobile phase. Rotational speed: 1600 rpm. Column temperature: 30 °C. Detection: 450 nm.

Once the operating parameters were optimized on the HPCCC analytical coil (24 mL), they were transferred to the HPCCC semi-prep coil (134 mL) to scale-up the developed method. The sample loading, flow rates and loop volume were scaled up linearly based on a principle previously established [56]. The “g” force in the analytical and semi-prep columns remained the same and the volume ratio between the analytical (24 mL) and semi-prep (134 mL) HPCCC coils was approximately 6. Accordingly, the sample loading, mobile phase flow rate and loop volume were adjusted proportionally to 240 mg (40 mg × 6), 3 mL/min (0.5 mL/min × 6) and 3 mL (0.5 mL × 6), respectively. These scale up settings led to a semi-prep chromatographic process (Figure 4a), showing a good stationary phase retention (*Sf*: 68.65%) at the hydrodynamic equilibrium stage. It was observed that fucoxanthin eluted at 37.2 min with a peak resolution of 2.5 with respect to the major contaminant and purity of 97%. However, it was noticed that a higher flow rate (4 mL/min) was still possible to produce fucoxanthin with a purity of 97% (Figure 4b) and a peak resolution of 2.3 with respect to the major contaminant. Therefore, these last conditions were selected in order to shorten the process and, thereby, increase the overall throughput and efficiency. In CCC, it is possible to predict the retention time of a given compound, once the stationary phase retention, partition coefficient value and flow rate are known. In the present study, it was calculated using the equation 2 as earlier described [57]. This information is of particular interest for calculating a priori the separation process duration and the solvent consumption. In the present study, the predicted retention time of fucoxanthin was 22.83 min, while its experimentally observed retention time was 28.45 min (Figure 4b). These values did not fully match up from each other, which may be due to a decrease in the stationary phase retention (*Sf*: 19.4%) during the separation process.

**Figure 5:**
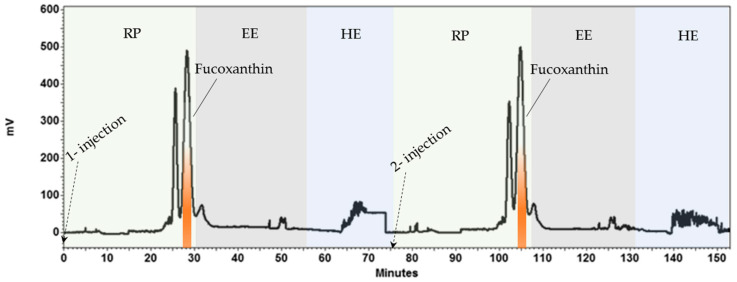
Development of two-injections high performance countercurrent chromatography (HPCCC) method to obtain fucoxanthin from *Phaeodactylum tricornutum* extract. Biphasic solvent system: System 1, mixture of *n*-heptane, ethyl acetate, ethanol and water (ratio 5:5:6:3, *v*/*v*/*v*/*v*). Elution modes: Reverse (RP) and elution-extrusion (EE). Hydrodynamic Equilibrium (HE). Loading per injection: 240 mg of *Phaeodactylum tricornutum* extract dissolved in 3 mL of mobile phase (3 mL sample loop). Runs: 2 consecutive injections. Flow rate: 4 mL/min. Rotational speed: 1600 rpm. Column temperature: 30 °C. Detection: 450 nm.

Next, in order to increase the productivity of the developed HPCCC separation process, a multiple-sequential injection system was applied combining two elution modes. It involves reverse phase elution mode for the fucoxanthin separation followed by elution-extrusion mode to replenish the column with new stationary phase without stopping the column rotation. After that, a new hydrodynamic equilibrium was achieved for a new separation cycle of fucoxanthin (Figure 5). The combination of these two HPCCC elution modes resulted as a feasible method for sequentially obtaining fucoxanthin from *P. tricornutum* biomass.

### 2.3. HPCCC Sequential Isolation of Fucoxanthin

**Figure 6:**
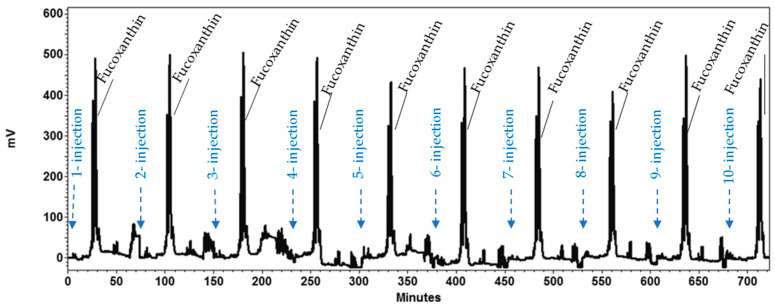
Multiple sequential injections—high performance countercurrent chromatography (HPCCC) method to obtain fucoxanthin from *Phaeodactylum tricornutum* extract. Biphasic solvent system: System 1, mixture of *n*-heptane, ethyl acetate, ethanol and water (ratio 5:5:6:3, *v*/*v*/*v*/*v*). Sample loading: 240 mg of extract dissolved in 3 mL of mobile phase (3 mL sample loop). Runs: 10 consecutive injections. Flow rate: 4 mL/min. Rotational speed: 1600 rpm. Column temperature: 30 °C. Detection: 450 nm.

**Figure 7:**
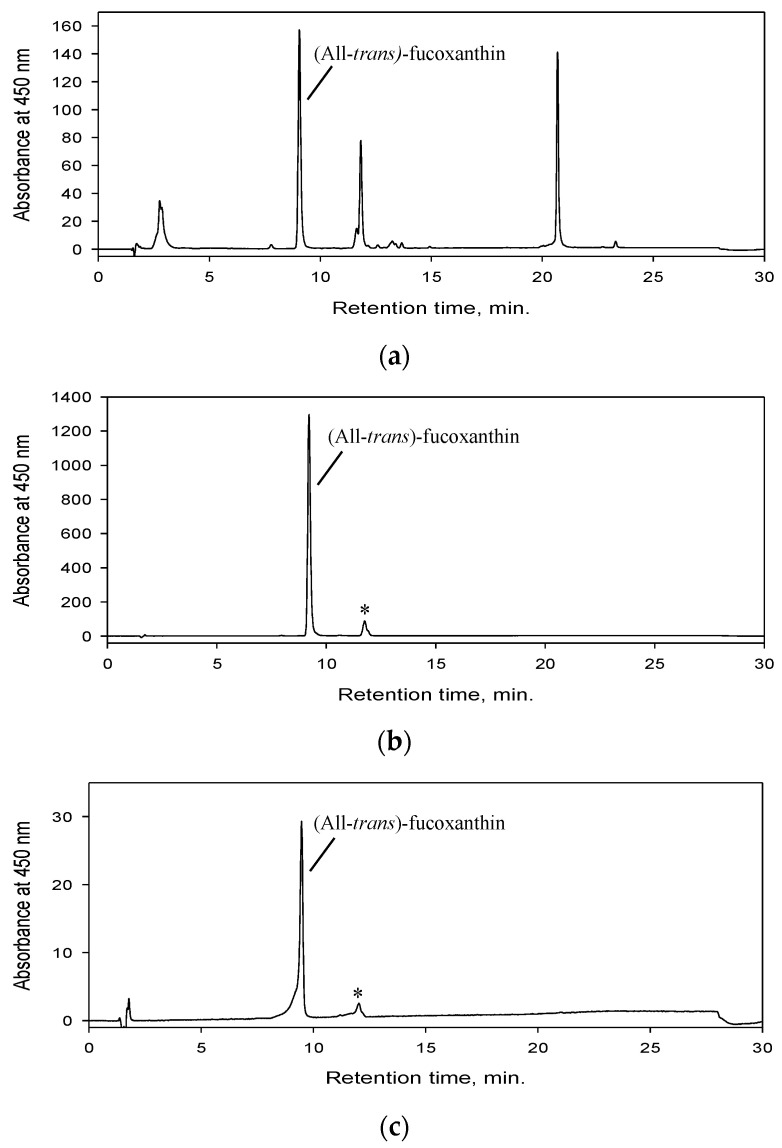
High performance liquid chromatography with diode array detection (HPLC-DAD) chromatograms of *Phaeodactylum tricornutum* extract (**a**), All-*trans*-fucoxanthin fraction obtained by high performance countercurrent chromatography (HPCCC) (**b**) and commercial standard of fucoxanthin from Sigma Aldrich (**c**). * (13 or 13′-*cis*)-fucoxanthin. The chromatograms were monitored at 450 nm.

Based on the previous optimized parameters and criteria, a sequential HPCCC separation process was satisfactorily performed to isolate fucoxanthin from *P. tricornutum* biomass (Figure 6). An amount of 240 mg of *P. tricornutum* extract was injected in each separation cycle. In total, 10 separation cycles were performed processing 2.4 g of *P. tricornutum* extract in 715 min. The entire HPCCC process took 762 min, which included a time period of 27 min for the first column filling with upper phase at 10 mL/min; 20 min for equilibration of the two liquid phases within the column by pumping lower phase at flow rate of 4 mL/min and followed by 715 min that comprised the ten separation cycles. Each separation cycle consisted of three steps including the separation of fucoxanthin in reversed phase elution mode (pumping lower phase at 10 mL/min for 31 min), extrusion (pumping upper phase at 10 mL/min for 25 min) and equilibration of liquid phases inside the column (pumping lower phase at 4 mL/min for 20 min). The chromatogram of the developed process is shown in Figure 6. The total separation process consumed 4.050 L of solvents, resulting in fucoxanthin (38 mg) with a purity of 97% (Figure 7b). To estimate the reproducibility of the separation process from run to run, the relative standard deviation (RSD) of the resolution values between the fucoxanthin and its major contaminant during the ten separation cycles process was calculated. It was found a RSD value of 7.54. Accordingly, the developed process showed a good reproducibility. Finally, as the content of fucoxanthin in the processed extract of *P. tricornutum* biomass was found to be 16.129 mg/g dried extract; therefore, the developed HPCCC process led to a fucoxanthin recovery of 98.16%.

### 2.4. Identity Confirmation of The Isolated Target Compound

**Figure 8:**
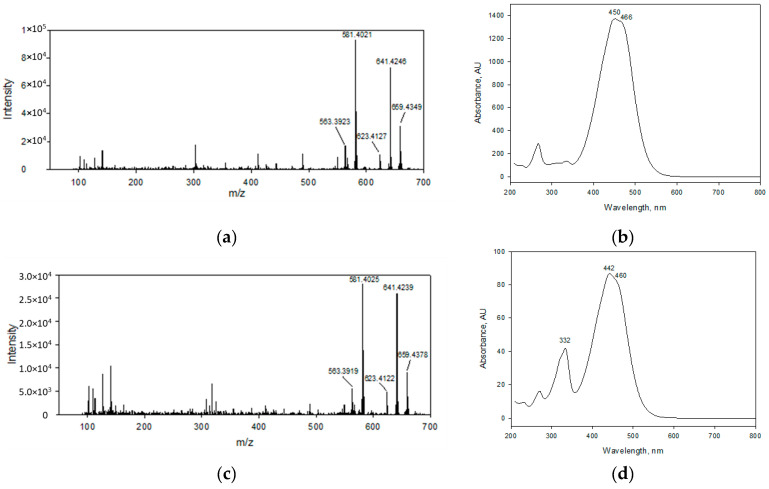
Atmospheric pressure chemical ionization-high resolution mass spectrometry (APCI-HRMS) of All-*trans*-fucoxanthin obtained by HPCCC (**a**) and (13 or 13′-*cis*)-fucoxanthin (**c**). Ultraviolet-visible (UV-Vis) spectra of All-*trans*-fucoxanthin obtained by HPCCC (**b**) and (13 or 13′-*cis*)-fucoxanthin (**d**).

The identity of the target compound was established as (All-*trans*)-fucoxanthin on the basis of its APCI-HRMS (Figure 8a) and UV-Visible (Figure 8b) spectra in comparison to the literature data [25,58]. The APCI-HRMS spectrum of the target compound peak displayed the molecular ion [M+H]^+^ at *m*/*z* 659.4349; a fragment ion [M+H-H_2_O]^+^ at *m*/*z* 641.4246 corresponding to the cleavage of a water molecule; a fragment ion [M+H-2H_2_O]^+^ at *m*/*z* 623.4127 formed by the loss of two water units; the ion with second-highest relative abundance [M+H-H_2_O-AcOH]^+^ was observed at *m*/*z* 581.4021 corresponding to the elimination of water and acetyl group from the molecular ion. The last ion at *m*/*z* 563.3923 [M+H-2H_2_O-AcOH]^+^ was generated by the cleavage of two water units followed by the dissociation of an acetyl group from the molecular ion. In the Figure 7b, one minor contaminant present in the fucoxanthin fraction obtained by means of HPCCC was observed, which showed an APCI-HRMS fragmentation pattern (Figure 8c) similar to that of (All-*trans*)-fucoxanthin (Figure 8a); therefore, they could not be distinguished from each other using mass spectrometry. The All-*trans*-fucoxanthin peak (Figure 7b) showed its typical UV-VIS spectrum (λmax: 450 and 466 nm) (Figure 8b), while the minor contaminant with retention time of 11.3 min was identified as a *cis*-fucoxanthin isomer based on its UV–VIS spectra (λmax: 442 and 460 nm together with characteristic band at 332 nm) (Figure 8d). This last compound is more likely to be 13- or 13′-*cis*-fucoxanthin, in line with its hypsochromic shift peculiarity and the intensity of the *cis* peak (D_B_/D_II_: 47.0%), as earlier published [59]. The *cis* isomers of carotenoids have been shown to be commonly generated from (All-*trans*)-carotenoids by light and temperature effects and constitute no risk to human health [45,47].

### 2.5. HPCCC Process Performance

Fucoxanthin has been mostly obtained from algal biomass using liquid-solid chromatography applying multi-step procedures [25,28,29,30,31,32,33]. Efforts have been dedicated to the production of fucoxanthin from algae using liquid-liquid chromatography namely CCC or CPC [50,51,52]; however, little has been done to improve the efficiency of the process for obtaining microalgae-sourced fucoxanthin. Unlike solid–liquid chromatography, no expensive columns are required in countercurrent chromatography [49,60]; thus, its use may represent a significant cost saving. The present study, copes with the challenge of applying a multiple-sequential injection strategy using HPCCC (Figure 6) for obtaining fucoxanthin from a microalgae diatom. The scale up of the developed isolation process can be performed in a volumetric way [56], as displayed in Table 3**.** The projected scaling up production of fucoxanthin from the lab scale (134 mL semi-prep column—Spectrum) to pilot size (18 L column—Maxi), maintaining the same "g" level, would enable processing 609.31 g of algal extract in less than a week. This demonstrates the scalability of the developed multiple-sequential injection system using HPCCC, which could cope with demand for large-scale production of fucoxanthin.

In this study, the developed separation process using a multiple-sequential injection strategy was aimed to enable the quick and large-scale isolation of fucoxanthin from the diatom *P. tricornutum*. Different liquid-liquid separation methods using CCC or CPC have already been reported for the separation of fucoxanthin from different algae species [50,51,52]. Two of these methods processed directly crude extracts using CPC for one microalgae species [52] and CCC for three macroalgae species [50]. A third reported method [51] involved a previous fractionation of macroalgal extract by solvent partitioning before CPC separation. Therefore, this last method [51] would not be useful for a comparative evaluation in this paper, as it uses a fucoxanthin-enriched fraction and would not ensure equality of experimental conditions, besides being an extensive method. Table 4 compares the process performance indicators (purity, *Pt*, *Pe*, *Er* and *Ge*) of the different methods. As shown in Table 4, most of the reported methods led to high purity fucoxanthin (90–99% pure). The purity of fucoxanthin produced in the present study was 97%. Although the different methods produced fucoxanthin with only slightly different purity values, they differed in the other process indicators. Due to the lack of some data referred to fucoxanthin separation in methods A [50] and B [52], some values had to be assumed to make a comparison possible. Studies describing the method A (it uses macroalgae species *L. japonica*, *U. pinnatifida* and *S. fusiforme*) and B (it uses microalgae *Tisochrysis lutea*) did not report solvent consumption for filling columns; therefore, we assumed two column volumes for column filling, as was also performed in the present study. The method described in the current paper used a 134 mL column volume, while methods A and B used 240 and 250 mL of column volumes, respectively. Unlike method B, method A does not report the flow rate of solvent used for column filling; thus, we assumed a flow rate of 30 mL/min, as was also applied in method B. In Table 4, the highest *Pt* values (methods A and B) were in the range from 0.22 and 12, which were higher than that obtained in this paper (0.19). However, the *Pe* value of the developed method (0.003) was about 6–31-fold higher than those of the method A and 1.15-fold higher than that in method B. The method developed in this paper showed the lowest environmental risk factor (*Er*) representing about 6.43–35.06-fold lower than the value indicated in the method A and 2.46-fold lower than that of the method B. This demonstrates the reduced environmental impact of the developed multiple-sequential injection method. It is well established that for production on an industrial scale, a higher efficiency of the process and a lower associated environmental risk factor are the main goals [61], which is achieved with as high a *Ge* as possible. In the present study, the *Ge* factor of the developed multiple-sequential injection HPCCC method was 36–1075-fold and 2.78-fold higher than that of methods A and B, respectively.

### 2.6. Induction of Nuclear Translocation of FOXO3 by Fucoxanthin

**Figure 9:**
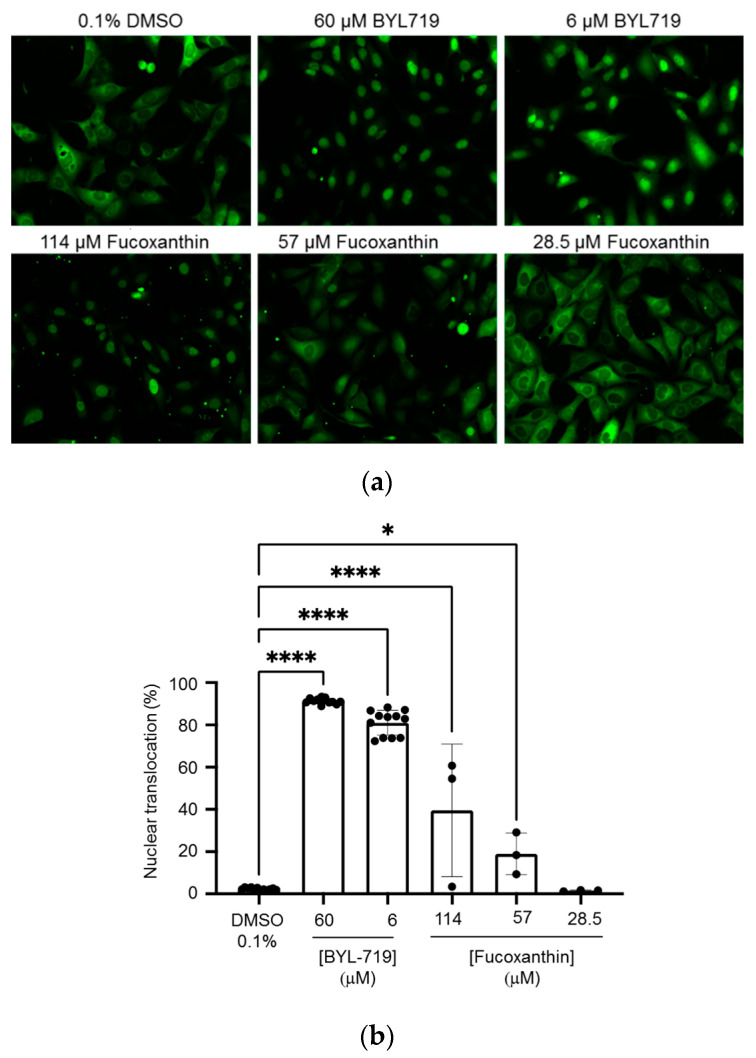
Induction of nuclear translocation of FOXO3 by *Phaeodactylum tricornutum*-derived fucoxanthin. (**a**) U2OS human osteosarcoma cell lines stably transfected with the chimeric construct FOXO3-GFP were treated with the isolated compound for 5 h and sub-cellular localization of the fusion protein was determined by confocal microscopy. (**b**) Quantification of the % values of nuclear translocation of the fusion protein FOXO3-GFP in the cells shown in (**a**). Bars represent the average of at least 3 replicates, indicated by dots. Error bars represent the standard deviation. Statistical significance was assessed using the one-way ANOVA with Tukey correction for multiple comparisons. *, *p* < 0.05; ****, *p* < 0.0001.

Fucoxanthin has been earlier described to induce FOXO3 nuclear translocation resulting in the reduction of oxidative stress and fibrosis in diabetic nephropathy [21,22]. A way to validate the viability of the developed multiple-sequential injection method using HPCCC for obtaining fucoxanthin is to check that the isolated compound retains its bioactivity. For these purposes, we tested the ability of the isolated fucoxanthin to act as an inductor of nuclear translocation of the transcription factor FOXO3, as already described [21,22]. In this bioassay, a well-described method based on the translocation of FOXO3 in the human osteosarcoma cell line U2OS [62,63,64] was used. As shown in Figure 9a,b, treatment of these cells for 5 h with the positive control (BYL-719) [65], induced a dose-dependent translocation of FOXO3 to the nucleus. Using this time of treatment, there was no evidence of viability decrease when cells were treated with fucoxanthin, as determined by cell number and morphology in the confocal assays. Previously described fucoxanthin-induced cell death in U2OS cells [66] was only apparent after a much longer treatment (48 h); and FOXO3 phosphorylation after treatment with fucoxanthin was measured 24 h after treatment [21]. Therefore, the FOXO3 nuclear translocation assay that is shown in the present study reports quick responses to treatments. Accordingly, compounds concentrations needed to elicit these quick responses (5 h) tend to be higher than those needed for long responses (24 or 48 h in previous literature). Importantly, treatment with the isolated fucoxanthin retained a clear ability to induce nuclear translocation of FOXO3 in a dose-dependent manner (Figure 9a,b). These results demonstrate that the isolated target compound not only shows good purity, but also retains bioactivity in an in vivo setting, thus proving the robustness and the chemically inert profile of the isolation method developed in this work.

## 3. Materials and Methods

### 3.1. Biomass Production

In this study, the microalgae *P. tricornutum* strain CCAP 1055/5 (Culture Collection of Algae and Protozoa—Scottish Association for Marine Science, Scotland, UK) was phototrophically cultivated in 80 L glass tubular photobioreactor. The culture was bubbled with a mixture of air and CO_2_ (98:2; *v*/*v*) at rate of 5 L/min to maintain a high turbulence in the reactor and to prevent cells sedimentation. The culture was continuously illuminated with a LED day light white lamp placed centrally inside the photobioreactor. Light intensity was set to 1500 μmol (photons) m^−2^ s^−1^ measured with a LI-250 light meter (LI-COR Biosciences, Lincoln, NE, USA) 3 cm above light body. The modified Artificial Seawater Medium [67] with added SiO3 solution (0.1 M) was used for cultivation at a temperature kept to 20 ± 1 °C. The cells were harvested by centrifugation using a refrigerated centrifuge (Sigma 8KS) at 20,461× *g* for 10 min. The resulting biomass was frozen to −70 °C and then lyophilized using a ScanVac CoolSafe freeze dryer (LaboGene ApS, Lynge, Denmark) for 72 h. An amount of 105 g of lyophilized *P. tricornutum* biomass was obtained. The microalgae growth curve is shown in Supplementary Figure S1.

### 3.2. Optimization of Biomass Extraction

For optimizing the production of fucoxanthin-enriched extract from *P. tricornutum* biomass, two extraction methods were investigated including ultrasound-assisted extraction (UAE) and mortar and pestle-assisted extraction (MPE). The tested extraction solvents were absolute ethanol, 80% ethanol (AnalaR Normapur, VWR Inc., Fontenay-sous-Bois, France), acetone, methanol (HiPerSolv Chromanorm, VWR Inc., Fontenay-sous-Bois, France), ethyl acetate, *n*-heptane (HiPerSolv Chromanorm, VWR Inc., Gliwice, Poland) as well as the upper and lower phases of the selected biphasic solvent system (Table 1). Bligh–Dyer method [68] was used as a reference procedure for extraction efficiency estimation. An amount of 10 mg of dried biomass was extracted with 5 mL of the corresponding solvent. UAE was performed for 10 and 30 min employing an ultrasound bath (K6 Kraintek, s.r.o., Podhájska, Slovakia) with a frequency of 38 kHz and an intensity of 47.7707 W/cm at 25 °C. High performance liquid chromatography with diode array detection (HPLC-DAD) was used for the determination of fucoxanthin content in the resulting extracts, as shown in Section 3.4. The best extraction system was used for the large-scale production of a fucoxanthin-enriched extract, from which pure fucoxanthin was obtained using HPCCC.

### 3.3. High Performance Countercurrent Chromatography (HPCCC) Separation

#### 3.3.1. HPCCC Equipment

For the isolation of fucoxanthin from *P. tricornutum* extract, a HPCCC equipment (Spectrum model, Dynamic Extractions Ltd., Slough, UK) equipped with a 134 mL column (PTFE bore tubing = 3.2 mm) was used. A speed regulator installed into the HPCCC chassis was used to control the speed of the HPCCC column. For controlling the column temperature, a H50/H150 Smart Water Chiller (LabTech Srl, Sorisole Bergamo, Italy) was used. To pump the mobile phase through the column, a Q-Grad pump (LabAlliance, State College, PA, USA) was used. The monitoring of the separation process was performed using a sapphire UV-VIS spectrophotometer (ECOM spol. s.r.o., Prague, Czech Republic) working at a wavelength of 450 nm. The separation process was simultaneously recorded by an EZChrom SI software platform (Agilent Technologies, Pleasanton CA, USA).

#### 3.3.2. Selection of the Suitable Biphasic Solvent System for HPCCC 

Several biphasic solvent systems were prepared using different volume ratios of *n*-heptane, ethyl acetate (HiPerSolv Chromanorm, VWR Inc., Gliwice, Poland), ethanol (AnalaR Normapur, VWR Inc., Fontenay-sous-Bois, France) and water. The obtained biphasic solvent systems were tested for their ability to be employed in HPCCC. A correct biphasic solvent system should provide a suitable partition coefficient (*K*) of the target compound [40,43], a proper density difference between its upper and lower phases and a short settling time [40,43]. The *K* value calculation was performed by dissolving 2 mg of the produced extract in 1 mL of each phase of the biphasic solvent systems. The obtained mixture was shaken and left to stand until the formation of two clear phases. The formed upper and lower phases were separated and used for the calculation of the *K* value using HPLC-DAD. The *K* value was calculated as the ratio between the fucoxanthin peak area in the upper phase to that of the lower phase [47,48]. The measurement of the settling time was performed as previously reported [47]. The density differences between the two immiscible phases were calculated by weighting 1 mL of each phase using a microbalance [43].

#### 3.3.3. HPCCC Separation Process

The HPCCC separation of fucoxanthin from *P. tricornutum* biomass was carried out using the selected biphasic solvent system composed of *n*-heptane, ethyl acetate, ethanol and water. The preparation of the solvent system was performed by mixing the individual amount of each solvent in a decanting funnel. Then, the mixture was vigorously shaken and left to stand until the formation of the two immiscible phases. The upper phase was employed as stationary phase and the lower phase as mobile phase. The HPCCC column was filled with the selected stationary phase and the rotational speed of the HPCCC column was set at 1600 rpm under a controlled temperature of 30 °C. Once the column was totally filled, the mobile phase was pumped through it until reaching the hydrodynamic equilibrium between the two immiscible phases within the HPCCC column. The equilibrium is achieved when the mobile phase front has emerged from the column without the carryover of the stationary phase; thus, at this steady stage, the system is ready for sample injection. The *P. tricornutum* extract dissolved in one volume of mobile phase was the sample solution. The HPCCC fractions were manually collected and analyzed by HPLC-DAD. 

The following equation was used to calculate the retention of the stationary phase (*Sf*) in the HPCCC column:

(1) $$Sf\;\left(\%\right)=\frac{Vs}{Vc}\times 100$$

where *V*c is the HPCCC column volume and *V*s is the stationary phase volume in the column when hydrodynamic equilibrium has been reached [57].

The following equation was used to predict the retention time (*t*_R_) of the target compound in the HPCCC separation:

(2) $$t_{R}=\frac{V_{M}+\left(K\times V_{S}\right)}{F}$$

where *V*_M_ is the mobile phase volume when the hydrodynamic equilibrium is reached, *K* is the partition coefficient of the target compound, *V*_S_ is the stationary phase volume when the hydrodynamic equilibrium has been reached and *F* is the mobile phase flow rate [57].

### 3.4. HPLC-DAD Analysis of Extract and Fractions

The *P. tricornutum* extract and HPCCC fractions were analyzed using a high performance liquid chromatography (HPLC) system (Agilent 1100 Series, Santa Clara, CA, USA) equipped with diode array detection (DAD). The chromatographic separation was performed using a reversed phase column (Luna^®^ C8 column, 100 × 4.6 mm, 3 μm, 100 Å) at 30 °C. The mobile phase consisted of the mixture of water (A) and methanol (B) which was pumped at a flow rate of 0.8 mL/min using a gradient elution as follows: 0–20 min, 20–0% A; 20–25 min, 0% A; 25–27 min, 0–20% A; 27–30 min, 20–20% A [47]. The HPLC analysis was monitored at 450 nm. A commercial standard of fucoxanthin was used for quantification and comparison purposes (Sigma Aldrich, Darmstadt, Germany). For estimating the content of fucoxanthin in microalgae extracts, a calibration curve was generated using five concentration points of the commercial standard of fucoxanthin ranging from 0.5 to 50 μg/mL, employing sample injection volumes of 20 μL. The resulting regression line equation was y = 126.3x (R^2^ = 0.9999), where x expresses fucoxanthin concentration (μg/mL) and y is the HPLC peak area. Purity of isolated fucoxanthin was determined using the same regression line equation.

### 3.5. Confirmation of the Chemical Identity of the Purified Target Compound 

The chemical identity of the isolated target compound was confirmed through a Dionex UltiMate 3000 HPLC system (Thermo Scientific, Carlsbad, CA, USA) connected to a high-resolution tandem mass spectrometry (HRMS/MS) detector with atmospheric pressure chemical ionization (APCI) source (Impact HD mass spectrometer Bruker, Billerica, MA, USA) (HPLC-APCI-HRMS) operated in positive ionization mode. Aiming to improve ionization efficiency, a formic acid (0.1%) solution was put in both solvents A and B. The MS operation parameters were set as follows: capillary voltage (2500 V), drying gas flow (5 L/min), drying gas temperature (350 °C), vaporizer temperature (450 °C) and nebulizer pressure (20 psi). The scanning of mass range between 100 and 2000 *m*/*z* was used for recording full-scan mass spectra. For the fragmentation of fucoxanthin, the collision energy was set to 35 eV and the collision gas was nitrogen. The identity of the target compound was confirmed in comparison with data published in literature. The conditions for the chromatographic separation are described in Section 3.4. 

### 3.6. High Performance Countercurrent Chromatography (HPCCC) Process Performance

The HPCCC process performance was evaluated as previously reported [61]. Four performance indicators were evaluated including process throughput (*Pt*), process efficiency (*Pe*), process environmental risk factor (*Er*) and general process evaluation factor (*Ge*).

The process throughput (*Pt*), which measures the mass processed per unit time, was calculated using the following equation:

(3) $$Pt=\frac{M_{c}}{t}$$

where *M_c_* is the mass of the algal extract injected per separation process and *t* is the time per separation process.

The process efficiency (*Pe*), which shows the rate of production of one mass unit of the isolated compound per unit time, was estimated using the following equation:

(4) $$Pe=\frac{M_{t}}{t}$$

where *M_t_* is the mass of the isolated target compound and *t* is the time consumption. 

Process environmental risk factor (*Er*), which shows the volume of the waste solvent generated in the production of one mass unit of isolated target compound, was estimated using the next equation:

(5) $$Er=\frac{V}{M_{t}}$$

where *V* is the total volume of solvent used in the process and *Mt* is the mass of the isolated target compound.

General process evaluation factor (*Ge*), which represents the process efficiency (*Pe*) relative to its environmental influence (*Er*), was determined using the following equation:

(6) $$Ge=\frac{Pe}{Er}$$

The indicators *Pt* and *Pe* were used to determine the potential productivity of the developed HPCCC process. The *Er* factor provided information on the environmental influence of the HPCCC process, and the *Ge* factor was used to show the overall performance of the entire HPCCC separation process in relation to its environmental impact. The higher the *Ge* factor, the more beneficial it will be.

### 3.7. Induction of Nuclear Translocation of FOXO3 

Induction of FOXO3 nuclear translocation was measured as previously described [62]. Briefly, U2OS human osteosarcoma-derived cells were stably transfected with the chimeric construct FOXO3-GFP [63]. Cells with the highest fluorescent intensity were sorted using an INFLUX^TM^ cell sorter (BD Biosciences, San Jose, CA, USA) and plated in 384-well plates. One day later, cells were treated with the indicated concentrations of compound and after 5 h, cells were fixed in 4% paraformaldehyde and stained with 1 µg/mL 40,6-Diamidino-2-phenylindole dihydrochloride (DAPI). Green fluorescence was measured using the high-throughput confocal microscope Opera LX (Perkin Elmer, Boston, MA, USA). Nuclear translocation was quantified using Acapella v2.0 (Perkin Elmer, Boston, MA, USA).

### 3.8. Statistical Analysis

The one-way ANOVA statistical test (*p* < 0.05) was used to determine difference among means followed by a Tukey’s multiple comparison test (*p* < 0.05) to perform pairwise comparisons. Relative standard deviation (RSD) of resolution values between the target compounds across the high performance countercurrent chromatography (HPCCC) separation was estimated. The data were analyzed using the Statistical Package S-Plus 2000. 

## Figures and Tables

**Table 1:** The partition coefficient (*K*) of fucoxanthin in different biphasic solvent systems, density differences and the settling times.

Solvent Systems	Composition	Relative Proportions of Solvents (*v*/*v*/*v*/*v*)	Phase Volume Ratio (UP/LP)	Settling Time (s)	Density Difference (LP−UP, g/mL)	Partition Coefficient (*K*) of Fucoxanthin
1	*n*-Hep–EtoAc–EtOH–H_2_O	5/5/6/3	0.58	17	0.1284	0.515
2	*n*-Hep–EtoAc–EtOH–H_2_O	5/5/7/3	0.43	15	0.1171	0.314
3	*n*-Hep–EtoAc–EtOH–H_2_O	5/5/8/3	0.38	18	0.1311	0.205
4	*n*-Hep–EtoAc–EtOH–H_2_O	5/5/6/4	0.59	18	0.1301	0.897
5	*n*-Hep–EtoAc–EtOH–H_2_O	5/5/6/5	0.59	20	0.1497	1.942
6	*n*-Hep–EtoAc–EtOH–H_2_O	5/5/5/3	0.80	20	0.1232	0.590

LP: Lower phase. UP: Upper phase.

**Table 2:** Stationary phase retention (*Sf*) and peak resolution in response to different sample loadings and flow rates in the analytical coil (24 mL) of high performance countercurrent chromatography (HPCCC) for obtaining fucoxanthin from *Phaeodactylum tricornutum* extract. Biphasic solvent system: System 1, mixture of *n*-heptane, ethyl acetate, ethanol and water (ratio 5:5:6:3, *v*/*v/v/v*). Elution mode: Reverse, the mobile phase is the lower phase of the biphasic solvent system. Column temperature: 30 °C. Detection: 450 nm. Loop volume: 0.5 mL.

Optimization Experiments	Flow Rate (mL/min)	*Sf* at The Hydrodynamic Equilibrium in HPCCC (%)	Loading Per Injection (mg)	Peak Resolution (1/2)	*Sf* at The End of The HPCCC Separation Run (%)	Peak Purity (%)
a	0.5	56.25	20	2.9	52.08	98
b	0.5	56.25	40	2.1	32.25	97
c	0.5	56.25	60	1.7	10.41	70
d	1.0	50	20	2.0	29.16	96
e	1.0	50	40	1.8	20.83	94
f	1.0	50	60	1.4	4.16	55

Rt: Retention time. The peak resolution was calculated as follows: Rs = 2(Rt_2_-Rt_1_)/(W_2_+W_1_)_._ (1) major contaminant. (2) fucoxanthin. W: the peak width at half height. a (Rt_1_ = 29 min, Rt_2_ = 38 min and W_1_~3.2, W_2_~3). b (Rt_1_ = 35 min, Rt_2_ = 43.4 min and W_1_~4.6, W_2_~3.35). c (Rt_1_ = 44.7 min, Rt_2_ = 49.4 min and W_1_~2.4, W_2_~3). d (Rt_1_ = 18.35 min, Rt_2_ = 21.9 min and W_1_~1.7, W_2_~1.9). e (Rt_1_ = 19.1 min, Rt_2_ = 22.5 min and W_1_~2.1, W_2_~1.75). f (Rt_1_ = 23 min, Rt_2_ = 24.9 min and W_1_~1.0, W_2_~1.7).

**Table 3:** Projected throughput for semi-preparative (used in this paper), preparative and pilot scale equipment for sequential (ten injections)—high performance countercurrent chromatography (HPCCC) process for obtaining fucoxanthin.

Equipment	Column Volume	Throughput	Throughput
Spectrum	134 mL	0.189 g/h	7.56 g/week ^a^
Midi	980 mL	1.382 g/h	55.28 g/week ^a^
Maxi	4.6 L	6.488 g/h	155.71 g/week ^b^
NSMS	8.820 L	12.440 g/h	298.56 g/week ^b^
Maxi	18 L	25.388 g/h	609.31 g/week ^b^

Throughput measured as mass of algal extract processed per unit time. New Spectrum modular series: NSMS. ^a^ Estimation assumes lab-scale equipment runs for 40 h/week. ^b^ Estimation assumes suitable equipment in the pilot plant and 24 h operation/week.

**Table 4:** Comparative results for different liquid-liquid separation methods.

HPCCC Process	Purity (%)	*Pt* (g/h)	*Pe* (g/h)	*Er* (L/g)	*Ge* (g^2^ h^−1^ L ^−1^)
Method in this paper	97.0	0.189	0.003	106.578	0.000028
Method A1 [50]	94.8	12.195	0.000405	900.6024	0.0000004496
Method A2 [50]	90.2	0.732	0.0005	685.7798	0.000000775
Method A3 [50]	90.4	7.317	0.0000976	3737.500	0.0000000261
Method B [52]	99.0	0.222	0.0026	261.797	0.00001010

*Pt*: Process throughput. *Pe*: Process efficiency. *Er*: Process environmental risk factor. *Ge*: General process evaluation factor. Estimation of *Pe* and *Er* uses the mass of isolated fucoxanthin. Methods A1, A2 and A3 use the macroalgae species *L. japonica*, *U. pinnatifida* and *S. fusiforme*, respectively.

## Supplementary Materials

The following are available online at https://www.mdpi.com/article/10.3390/md19090517/s1, Figure S1: Growth curve of *Phaeodactylum tricornutum*.
