# Supplementary material for: Production of Fucoxanthin from Phaeodactylum tricornutum Using High Performance Countercurrent Chromatography Retaining Its FOXO3 Nuclear Translocation-Inducing Effect
Source: Mar Drugs. 2021 Sep 11;19(9):517. doi: 10.3390/md19090517 (PMC8466784; doi:10.3390/md19090517)

Figure S1. Growth curve of *Phaeodactylum tricornutum* P5-CCAP in 80 L tubular photobioreactor. The culture growth was monitored by V-1200 spectrophotometer as optical density at near infrared wavelength 750 nm with optical path length 5 mm.

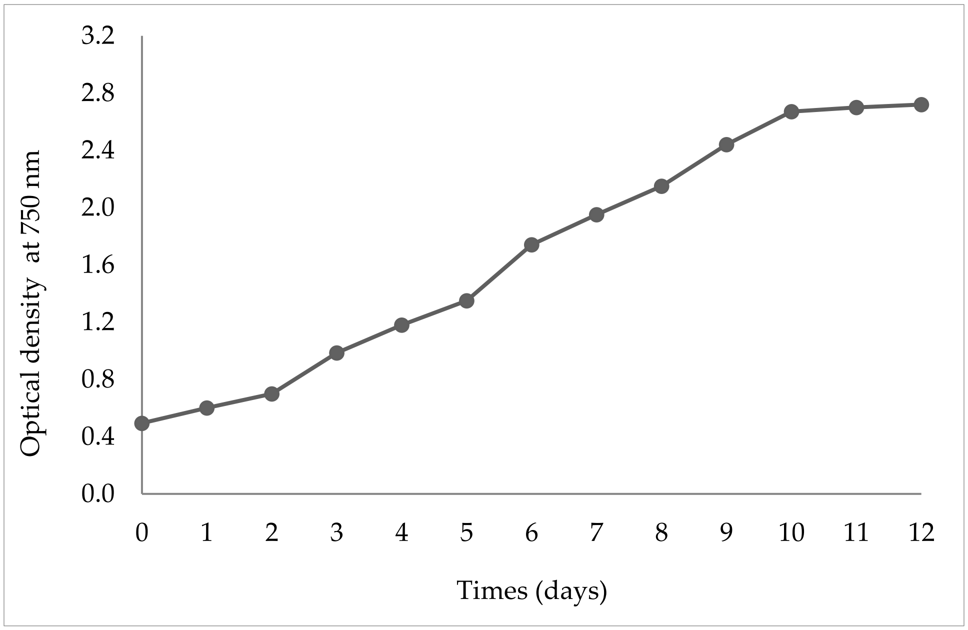

Supplement: Supplementary file 1 [file marinedrugs-19-00517-s001.zip › marinedrugs-1337395-supplementary.pdf]
